# Endobronchial mass in an elderly smoker

**DOI:** 10.4103/1817-1737.58962

**Published:** 2010

**Authors:** Roman Dutta, Ghan Shyam Pangtey, Ruchika Gupta, Arvind Kumar

**Affiliations:** *Department of Surgical Disciplines, All India Institute of Medical Sciences, New Delhi-110 029, India*; 1 *Department of Medicine, All India Institute of Medical Sciences, New Delhi-110 029, India*; 2 *Department of Pathology, All India Institute of Medical Sciences, New Delhi-110 029, India*

A 65-year-old man presented with complaints of cough with minimal mucoid expectoration and wheezing for 2 months. He was a chronic smoker with a smoking index of 30 pack-years. He had marked anorexia, with weight loss of 5 kg over 2 months. There was no history of fever, chest pain, or hemoptysis. He was not a hypertensive or diabetic, and there was no history of visits to the emergency room for acute respiratory distress or exacerbation of dyspnea. There was no history of diarrhea, flushing, or abdominal pain.

He was of average build and nutrition. There was no clubbing of the fingers or any palpable peripheral lymph node. He had slight drooping of the shoulder on the right side. On auscultation, a monophonic wheeze was heard in the right mammary area, with normal vesicular breath sounds present over the rest of the chest. Cardiovascular and abdominal examination was unremarkable. Laboratory evaluation revealed hemoglobin of 10.5 gm%, total leukocyte count (TLC) of 5600/dl, and platelet count of 2.2 × 10^5^/dl. His ESR in the first hour was 30 mm (Westergren method). He had normal renal and liver function tests. Three consecutive sputum examinations were negative for malignant cells as well as for acid-fast bacilli (AFB). Mantoux skin test done over the left forearm was positive, with induration of 15 mm after 48 h. His chest radiograph revealed right hilar prominence, without any lung parenchyma abnormalities [Figure 1]. A contrast-enhanced computed tomography (CECT) scan of the chest showed an endobronchial mass in the right main bronchus, with narrowing of the lumen [Figure 2]. There was right hilar lymphadenopathy [Figure 3]. Flexible fiberoptic bronchoscopy (FOB) confirmed the CECT findings of a nodular growth in the right main bronchus with surrounding mucosal edema and narrowing of the lumen. Multiple biopsies were taken from the nodular mass.

**Figure 1:**
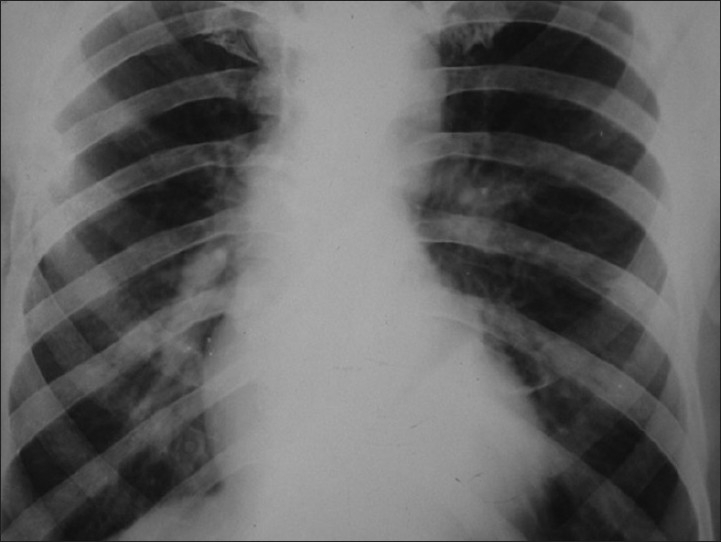
Chest radiograph showing right hilar prominence

**Figure 2:**
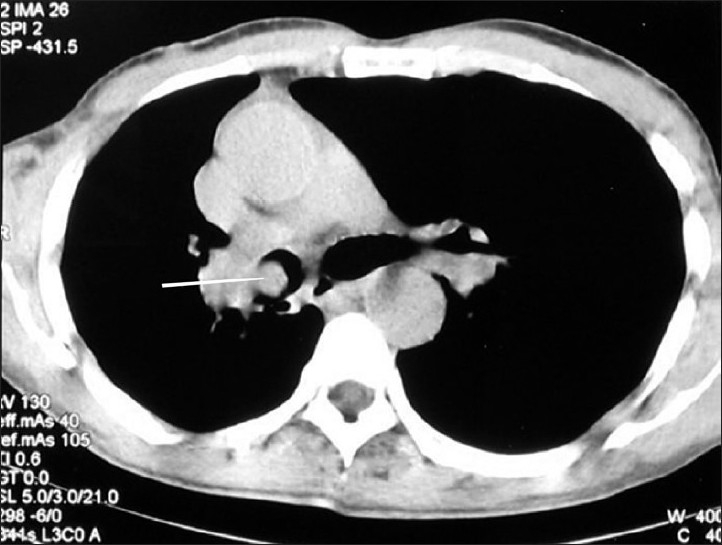
CECT chest axial section in mediastinal window, showing nodular lesion inside the right main bronchus (arrow)

**Figure 3:**
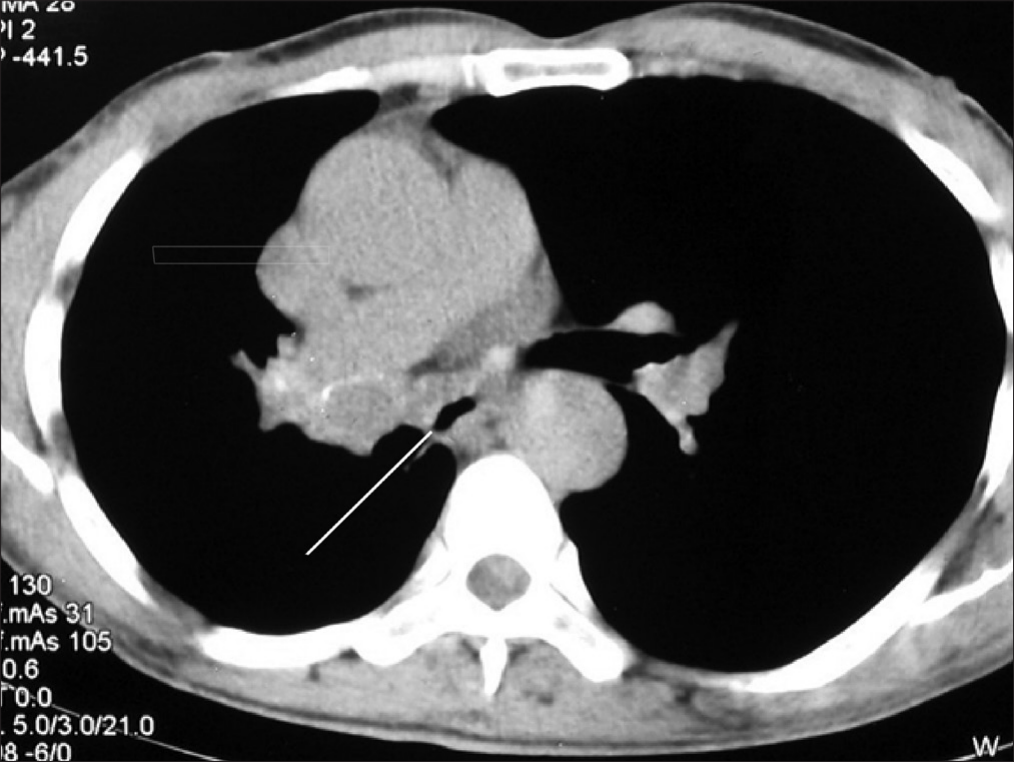
CECT chest axial section at a lower level showing hypodense right Hilar lymphadenopathy with narrowing of bronchus intermedius (arrow)

## Clinical Questions

Based on the clinical history and investigation results, what is your provisional diagnosis?

What will be your approach if you have an inconclusive result from the histopathological examination of the biopsy specimen from the growth?

## Answers

A provisional diagnosis of bronchogenic carcinoma was first considered in view of the patient's age and smoking history and the radiological and bronchoscopy findings of an endobronchial growth. The other differential diagnoses considered were bronchial carcinoid, adenoma, hamartoma, adenoid cystic tumor, lipoma, and an infectious lesion. Histological examination of the biopsy material from the growth was performed to get a conclusive diagnosis.

**Figure 4:**
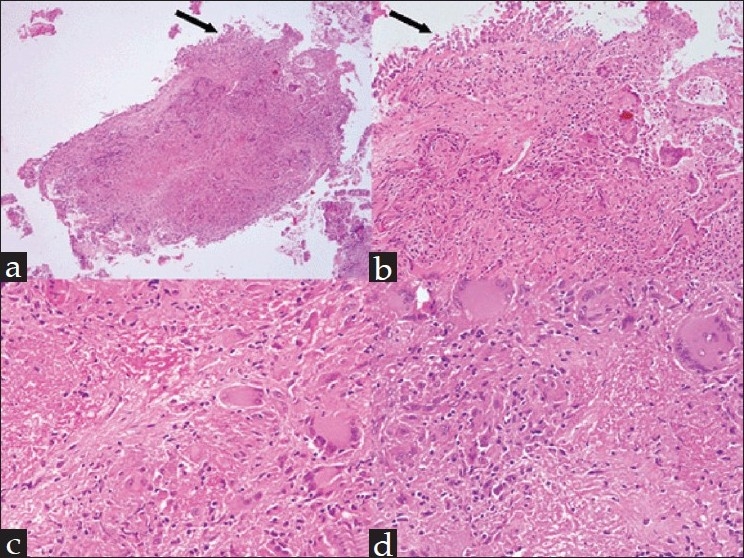
Photomicrograph showing bronchial epithelium (black arrow) and subepithelial granulomatous inflammation (a and b, H and E, ×4 and ×10, respectively). High-power view of the granuloma showing caseous necrosis surrounded by epitheloid cells and Langhans giant cells (c and d, H and E, ×20).

It is not unusual to have an inconclusive histopathological result from the FOB biopsy specimen. It would be wise to repeat the FOB when there is an inconclusive histopathological result. The FOB biopsy of the endobronchial mass in this case was suggestive of tuberculosis [Figure 4]. Based on the computed tomographic (CT) features and the FOB and histopathologic findings, a diagnosis of endobronchial tuberculosis (EBTB) was made and four-drug antitubercular treatment (ATT) was started, with rifampicin, isoniazid, pyrazinamide, and ethambutol (ERHZ) for the first 2 months, followed by a continuation phase of 4 months with isoniazid and rifampicin (WHO category 1 treatment). Within 2 months the patient's appetite improved and he gained weight. He completed the full course of ATT and had an uneventful recovery.

## Discussion

EBTB is an uncommon subtype of pulmonary tuberculosis and is defined as tubercular infection of the tracheobronchial tree with microbial and histopathological evidence.[1] In the pre-ATT era, EBTB was seen in 10–15% of patients.[2] However, with the advent of ATT, the incidence of EBTB is rapidly decreasing. The first case was described by Morten *et al*.[3] EBTB may be associated with bronchial stenosis as it frequently heals with concentric scarring; therefore, it may lead to secondary collapse and consolidation.[4]

Tuberculosis is a very common disease in the tropics and has varied presentations, ranging from pulmonary parenchymal involvement to extrapulmonary disease. However, endobronchial infection is a relatively uncommon manifestation of this common disease in the post-ATT era. There are only isolated case reports in the English literature on the clinical, bronchoscopic, and radiologic features of EBTB.[5] On the basis of FOB findings, EBTB has been classified into seven subtypes: actively caseating, edematous–hyperemic, fibrostenotic, tumorous, granular, ulcerative, and nonspecific bronchitis.[5] Five different mechanisms have been postulated to explain the spread of endobronchial tuberculosis, viz. direct extension from an adjacent focus in the lung parenchyma; implantation of organisms from the infected sputum; hematogenous dissemination; lymph node erosion into the bronchus; and through the lymphatic drainage, from the parenchyma to the peribronchial region.[6]

EBTB is highly infectious and is present in 10–40% of patients with active pulmonary tuberculosis.[7] Although it is more common in young females, up to 15% of geriatric patients have also been reported to be affected with EBTB in different studies.[8] In the elderly patient it may mimic malignancy or a nonresolving pneumonia and this may be one of the reasons for underestimation of EBTB. Cough is usually the main symptom, followed by chest pain and wheeze. Hemoptysis may be present in addition to the constitutional symptoms of tuberculosis. Our patient had a chronic dry cough and a monophonic wheeze, associated with the systemic symptoms of weight loss and anorexia. In a published series of bronchoscopic findings in the elderly, four out of seven patients with EBTB were suspected to have malignancy on FOB.[9] In our case also, the diagnosis of EBTB was made based on histopathological examination after bronchoscopic biopsy of the endobronchial mass.

The usual treatment of EBTB is with four-drug ATT. Our patient was treated with this regimen for 6 months and he improved clinically, with complete resolution of the radiological lesions in the CECT scan of the chest. The role of oral and nebulized steroids, and of aminoglycosides, is still controversial.[10]

The early diagnosis and prompt treatment of EBTB prevents complications such as disseminated disease or bronchial stenosis. This case highlights the importance of considering common and curable conditions like tuberculosis in the differential diagnosis of an endobronchial growth or mass and stresses the fact that tissue diagnosis should be undertaken in all cases before the mass is labeled as malignant.
